# Machine Learning–Based Prediction of Suicidal Thinking in Adolescents by Derivation and Validation in 3 Independent Worldwide Cohorts: Algorithm Development and Validation Study

**DOI:** 10.2196/55913

**Published:** 2024-05-17

**Authors:** Hyejun Kim, Yejun Son, Hojae Lee, Jiseung Kang, Ahmed Hammoodi, Yujin Choi, Hyeon Jin Kim, Hayeon Lee, Guillaume Fond, Laurent Boyer, Rosie Kwon, Selin Woo, Dong Keon Yon

**Affiliations:** 1 Center for Digital Health Medical Science Research Institute Kyung Hee University College of Medicine Seoul Republic of Korea; 2 Department of Applied Information Engineering, Yonsei University Seoul Republic of Korea; 3 Department of Precision Medicine Kyung Hee University College of Medicine Seoul Republic of Korea; 4 Department of Regulatory Science Kyung Hee University Seoul Republic of Korea; 5 Division of Sleep Medicine, Harvard Medical School Boston, MA United States; 6 Department of Anesthesia, Critical Care and Pain Medicine, Massachusetts General Hospital Boston, MA United States; 7 Department of Business Administration, Kyung Hee University School of Management Seoul Republic of Korea; 8 Department of Korean Medicine, Kyung Hee University College of Korean Medicine Seoul Republic of Korea; 9 Assistance Publique–Hôpitaux de Marseille (APHM), CEReSS–Health Service Research and Quality of Life Center, Aix–Marseille University Marseille France; 10 Department of Pediatrics Kyung Hee University Medical Center Kyung Hee University College of Medicine Seoul Republic of Korea

**Keywords:** adolescent, machine learning, Shapley additive explanations, SHAP value, suicidal thinking, XGBoost, mental health, predictive model, risk behavior

## Abstract

**Background:**

Suicide is the second-leading cause of death among adolescents and is associated with clusters of suicides. Despite numerous studies on this preventable cause of death, the focus has primarily been on single nations and traditional statistical methods.

**Objective:**

This study aims to develop a predictive model for adolescent suicidal thinking using multinational data sets and machine learning (ML).

**Methods:**

We used data from the Korea Youth Risk Behavior Web-based Survey with 566,875 adolescents aged between 13 and 18 years and conducted external validation using the Youth Risk Behavior Survey with 103,874 adolescents and Norway’s University National General Survey with 19,574 adolescents. Several tree-based ML models were developed, and feature importance and Shapley additive explanations values were analyzed to identify risk factors for adolescent suicidal thinking.

**Results:**

When trained on the Korea Youth Risk Behavior Web-based Survey data from South Korea with a 95% CI, the XGBoost model reported an area under the receiver operating characteristic (AUROC) curve of 90.06% (95% CI 89.97-90.16), displaying superior performance compared to other models. For external validation using the Youth Risk Behavior Survey data from the United States and the University National General Survey from Norway, the XGBoost model achieved AUROCs of 83.09% and 81.27%, respectively. Across all data sets, XGBoost consistently outperformed the other models with the highest AUROC score, and was selected as the optimal model. In terms of predictors of suicidal thinking, feelings of sadness and despair were the most influential, accounting for 57.4% of the impact, followed by stress status at 19.8%. This was followed by age (5.7%), household income (4%), academic achievement (3.4%), sex (2.1%), and others, which contributed less than 2% each.

**Conclusions:**

This study used ML by integrating diverse data sets from 3 countries to address adolescent suicide. The findings highlight the important role of emotional health indicators in predicting suicidal thinking among adolescents. Specifically, sadness and despair were identified as the most significant predictors, followed by stressful conditions and age. These findings emphasize the critical need for early diagnosis and prevention of mental health issues during adolescence.

## Introduction

Adolescent suicide stands out as a prominent global public health concern, with its rank as the second leading cause of death among young populations underscoring its severity [1,2] Notably, adolescence is a phase characterized by an amplified suicide risk [3]. Concerningly, some geographic regions are experiencing a surge in suicide clusters, where the instances of suicide exceed the typical levels [4]. Research into these clusters indicates that individuals younger than 25 years are up to 4 times more likely to be affected by suicide [5]. Since suicide is preventable in the early stages, there is a pressing need for action through rigorous mental health strategies and proactive educational interventions [6].

While various methodologies have been proposed to prevent suicidal thinking in adolescents, many lack empirical outcomes and often fail to identify key determinants [7-9]. A significant gap remains in accurately assessing the risk of suicidal thinking for individual adolescents [2,10,11]. Recent advances in machine learning (ML) methodologies have shown promise in addressing the challenges of adolescent suicidal tendencies. Studies leveraging boosted ML [12], daily data analysis through classification and regression trees [13], and risk and protective factor frameworks [14] have begun to unpack the complex interplay of factors contributing to suicidal thinking among adolescents. However, these studies have also highlighted limitations, including a focus on specific socioeconomic or short-term predictors and a lack of comprehensive risk profiles integrating emotional, social, and psychological variables [12-14].

Therefore, in this study, we developed a predictive model for suicidal thinking among adolescents, using advanced ML algorithms. Addressing the gaps identified in earlier research, our model incorporates a broader array of factors, including family dynamics, emotional well-being, academic performance, and general health indicators. Across distinct adolescent cohorts from South Korea, Norway, and the United States, we aimed for a comprehensive multinational approach. By refining our approach based on previous studies’ insights, this research aims to highlight the preventability of suicide and influence mental health clinicians and policy makers to develop more effective preventive measures and supportive programs.

## Methods

### Study Design and Participants

This study aimed to develop an ML model to predict suicidal thinking among Korean adolescents. Our approach used multiple variables extracted from 3 distinct, large-scale international data sources: the Korea Youth Risk Behavior Web-based Survey (KYRBS) [15,16], the Youth Risk Behavior Survey (YRBS), and Norway’s nationwide University National General Survey (Ungdata).

### Data Preparation and Harmonization

Initial data preprocessing involved adjusting the sample sizes after the removal of missing values: KYRBS from 1,145,178 to 566,875, YRBS from 438,566 to 103,874, and Ungdata from 89,077 to 19,574. We analyzed data from adolescents aged between 13 and 18 years who participated in the KYRBS from 2009 to 2021, the YRBS in 2021, and Ungdata from 2017 to 2019. The primary outcome, termed “current suicidal thinking,” was derived from participants’ affirmative responses to the question, “During the past 12 months, did you ever seriously consider attempting suicide?” This outcome indicated that participants had contemplated serious suicidal thinking at least once in the preceding year. The analysis considered several covariates: region, age, sex, BMI (kg/m^2^), academic achievement, household income, smoking status, alcoholic consumption, stress status, feelings of sadness and despair, exercise habits, and screen time (Figure S1 in Multimedia Appendix 1) [17].

We harmonized the data sets for XGBoost model compatibility, addressing the challenge posed by different variable configurations within the same questions. Our preprocessing aligned each variable across the KYRBS, YRBS, and Ungdata data sets, ensuring they matched in terms of content and format. Recognizing the potential disparities in variable configurations across these data sets, we standardized the variable names, formats, and scales, focusing on key features such as demographic information, behavioral factors, psychosocial aspects, and environmental influences that could serve as predictors for suicidal thinking. To ensure consistency, we adopted the following strategic approach to cases where YRBS and Ungdata were missing certain features: by calculating the median of the missing variables in the KYRBS data set, we were able to effectively impute the missing values to maintain the integrity and comparability of the data set compilations. Through variable alignment and addressing missing data, we successfully harnessed the diverse strengths of each data set, facilitating the development of a comprehensive model designed to address adolescent suicidal thinking effectively.

### ML Model Development

Our ML model underwent training and validation to ensure its predictive accuracy in identifying suicidal thinking. We used the KYRBS data set to build a model tailored to predict suicidal thinking among Korean adolescents aged between 13 and 18 years. Recognizing the intricate characteristics of the data, we used a variety of tree-based ML techniques, including XGBoost, adaptive boosting (AdaBoost), light gradient-boosting machine (LightGBM), and random forest to train the data set for our modeling process [18]. Before this, data preprocessing measures, such as addressing missing values and encoding categorical variables, were executed to maintain data integrity and optimize the data for the modeling phase.

We adopted the 10-fold cross-validation method, dividing the initial data set into 10 equal-sized subsets, to rigorously assess the performance efficacy of the ML model. Of these, 9 are designated for model training, while the remaining subset serves as validation [19]. The process iterates 10 times, ensuring each subset undergoes validation at least once. During each cycle, we computed various performance metrics such as area under the receiver operating characteristic (AUROC) curve, sensitivity, specificity, accuracy, and balanced accuracy, along with their respective 95% CIs [20-25]. The 95% CIs provide a range of possible values for the model’s performance metrics, allowing us to assess the stability and generalizability of the model. For a visual representation of the model efficacy, we used visualization methods, primarily the receiver operating characteristic curve. After 10 iterations, the metrics from each were averaged to determine the final performance evaluation.

We trained our model on the KYRBS data set using 10-fold cross-validation. The model trained on the KYRBS data set was then externally validated with YRBS and Ungdata data sets preprocessed with the same column structure as KYRBS. This rigorous process reinforced the reliability of our model’s performance trained on the KYRBS data set [26]. Among the 4 models tested, XGBoost consistently yielded the highest AUROC scores across all data sets, leading to its selection as the primary model.

We performed hyperparameter tuning using GridSearchCV to optimize the performance of the XGBoost model, prioritizing the maximization of the AUROC score to determine the optimal hyperparameter combination. Hyperparameters were carefully selected for improved performance: the gbtree booster was used for its effectiveness in classification tasks, and the logloss evaluation metric was chosen to ensure accurate probability estimations. We set the learning rate at 0.08 to balance training speed with model accuracy, and the max depth was capped at 5 to prevent overfitting while allowing the model to capture complex patterns. Additionally, 350 trees (n_estimators) were used to construct a robust model, with further adjustments made to parameters like “scale_pos_weight” and subsample to address class imbalance and enhance model stability. These adjustments were important in refining our model’s predictive capabilities and are detailed in Table S1 in Multimedia Appendix 1. In order to interpret and gain insights into the model predictions, we used Shapley additive explanations (SHAP) values, a unified measure derived from cooperative game theory. Data set variables were analyzed with SAS software (version 9.3; SAS Institute Inc), and ML analysis was performed using Python (version 3.11.4; Python Software Foundation). The main Python libraries used are as follows: NumPy (version 1.26.0; Python Software Foundation) for data arrays and operations, and Pandas (version 2.1.0; Python Software Foundation) for data manipulation and analysis. All 3—scikit-learn (version 1.2.2; scikit-learn development team), TensorFlow-gpu (version 2.6.0; Tensor development team), and Keras (version 2.6.0; Keras development team)—were used for constructing and training ML models [27]. Additionally, the SHAP package (version 0.42.1) was used to interpret the ML models and for its explanation capabilities [28].

### Software and Libraries

All computations, model training, and evaluations were executed using Python (version 3.11.4). Key libraries from our toolbox included scikit-learn (version 1.2.2), NumPy (version 1.24.0), and Pandas (version 2.1.0) for ML tasks and data wrangling. Visualization was facilitated using Matplotlib (version 3.7.2) and Seaborn (version 0.12.2).

### Ethical Considerations

The study protocol was approved by the institutional review board of the Korean Disease Control and Prevention Agency (2014-06EXP-02-P-A), the US Centers for Disease Control and Prevention (#1969.0), the Norwegian Centre for Research Data and Data Protection Office of Inland Hospital Trust (18778329) and by the local law of the Population Health Promotion Act 19 (117058) form of the Korean government. All participants provided written informed consent. This research followed the guidelines outlined in the TRIPOD (Transparent Reporting of a Multivariable Prediction Model for Individual Prognosis or Diagnosis) statement (Table S2 in Multimedia Appendix 1).

## Results

### Demographic Characteristics

**Figure 1:**
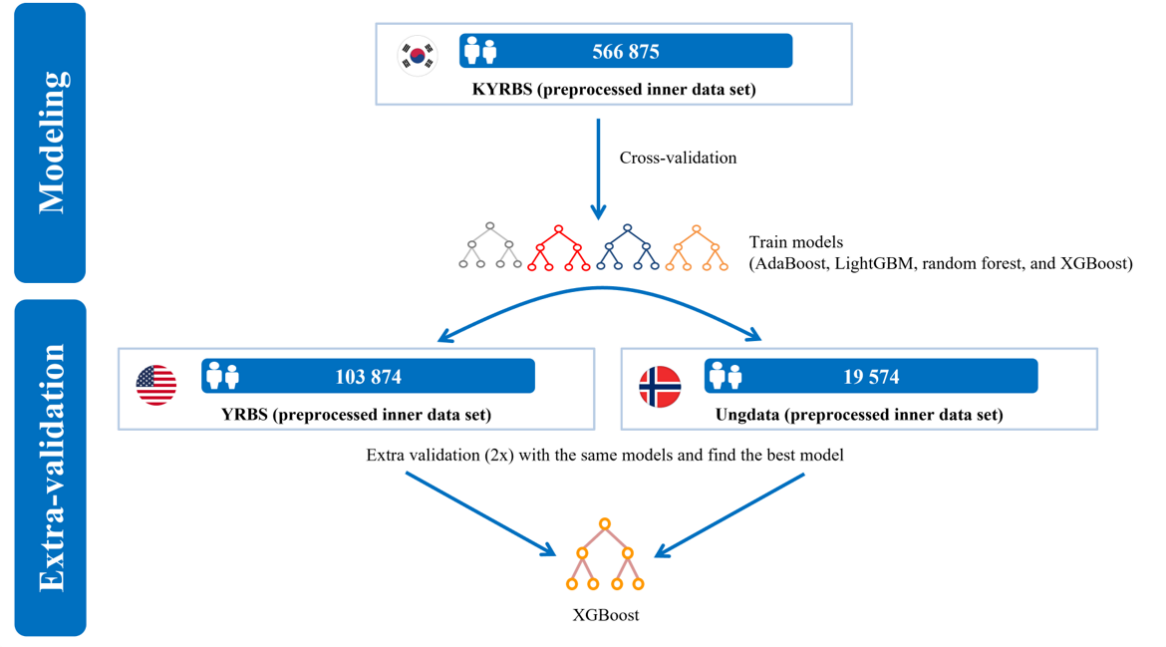
Study architecture. AdaBoost: adaptive boosting; KYRBS: Korea Youth Risk Behavior Web-based Survey; LightGBM: light gradient-boosting machine; Ungdata: Norwegian nationwide Ungdata surveys; YRBS: Youth Risk Behavior Survey.

**Figure 2:**
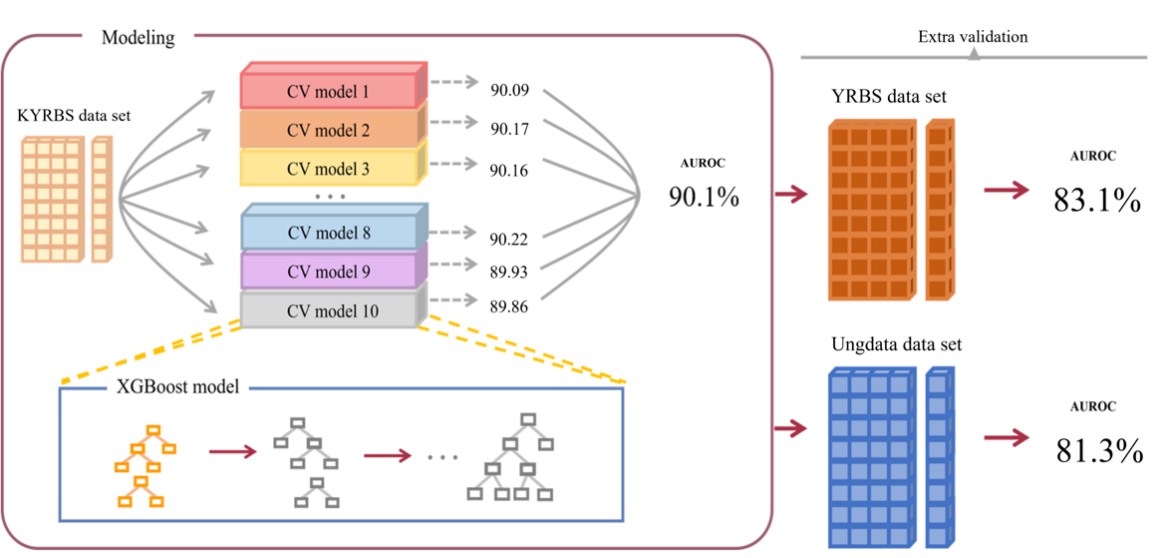
Model architecture. The original Korea Youth Risk Behavior Web-based Survey (KYRBS) data set was partitioned into the original data set for model development, with performance assessed using area under the receiver operating characteristic (AUROC) curve scores. Selected high-performing models were further validated using an external Youth Risk Behavior Survey (YRBS) data set. The validation results were derived from the original data set, external results from the additional YRBS data set, and the Norwegian nationwide Ungdata survey (Ungdata) data set. CV: cross validation.

This study was conducted to develop a ML-based predictive model for suicidal thinking among adolescents aged between 13 and 18 years. After collecting independent data from 3 countries, covariates were standardized for the ML prediction modeling process (Figures 1 and 2).

The distribution of age in the initial training cohort for KYRBS from South Korea, which was used to build the prediction model, was as follows: aged 13 years (94,923/566,875, 16.74%), aged 14 years (98,624/566,875, 17.4%), aged 15 years (100,490/566,875, 17.73%), aged 16 years (90,057/566,875, 15.89%), aged 17 years (92,071/566,875, 16.24%), and aged 18 years (90,710/566,875, 16%). For the external validation cohort using YRBS from the United States, the age distribution was as follows: aged 13 years (351/103,874, 0.34%), aged 14 years (21,095/103,874, 20.31%), aged 15 years (28,016/103,874, 26.97%), aged 16 years (25,929/103,874, 24.96%), aged 17 years (22,405/103,874, 21.57%), and aged 18 years (6078/103,874, 5.85%). Another external validation stage using Ungdata from Norway had the following age distribution: aged 13 years (5039/19,574, 25.74%), aged 14 years (4874/19,574, 24.9%), aged 15 years (5034/19,574, 25.72%), aged 16 years (3181/19,574, 16.25%), aged 17 years (845/19,574, 4.32%), and aged 18 years (601/19,574, 3.07%) (Table 1).

**Table 1:** Demographic characteristics of Korea Youth Risk Behavior Web-based Survey (KYRBS) data from South Korea (2009-2021), Youth Risk Behavior Survey (YRBS) data from the United States (2021), and University National General Survey (Ungdata) from Norway (2017-2019).

Characteristics											KYRBS (n=566,875)		YRBS (n=103,874)	Ungdata (n=19,574)
**Region, n (%)**														
									Urban		259,453 (45.77)		N/A^a^	N/A
									Rural		307,422 (54.23)		N/A	N/A
**Age (years), n (%)**														
							13				94,923 (16.74)		351 (0.34)	5039 (25.74)
							14				98,624 (17.4)		21,095 (20.31)	4874 (24.9)
							15				100,490 (17.73)		28,016 (26.97)	5034 (25.72)
							16				90,057 (15.89)		25,929 (24.96)	3181 (16.25)
							17				92,071 (16.24)		22,405 (21.57)	845 (4.32)
							18				90,710 (16)		6078 (5.85)	601 (3.07)
**Sex, n (%)**														
	Male									289,311 (51.04)		51,842 (49.91)		9812 (50.13)
	Female									277,564 (48.96)		52,032 (50.09)		9762 (49.87)
**BMI^b^, n (%)**														
						Underweight					44,539 (7.86)		12,541 (12.07)	N/A
						Normal					423,384 (74.69)		47,592 (45.82)	N/A
						Overweight					48,951 (8.64)		14,439 (13.9)	N/A
						Obese					50,001 (8.82)		29,302 (28.21)	N/A
**Academic achievement, n (%)**														
								Low (0-19th percentiles)			56,799 (10.02)		N/A	N/A
								Lower-middle (20th -39th percentiles)			132,774 (23.42)		N/A	N/A
								Middle (40th-59th percentiles)			162,447 (28.66)		N/A	N/A
								Upper-middle (60th-79th percentiles)			146,401 (25.83)		N/A	N/A
								High (80th-100th percentiles)			68,454 (12.08)		N/A	N/A
**Household income, n (%)**														
					Low (0-19th percentiles)					19,200 (3.39)		N/A		8466 (43.25)
					Lower-middle (20th -39th percentiles)					82,314 (14.52)		N/A		6557 (33.5)
					Middle (40th-59th percentiles)					274,093 (48.35)		N/A		3523 (18)
					Upper-middle (60th-79th percentiles)					148,848 (26.26)		N/A		818 (4.18)
					High (80th-100th percentiles)					42,420 (7.48)		N/A		210 (1.07)
**Smoking status, n (%)**														
							Nonsmoker				467,707 (82.51)		84,984 (81.81)	16,594 (84.78)
							Smoker				99,168 (17.49)		18,890 (18.19)	2980 (15.22)
**Alcohol consumption, n (%)**														
		Nondrinker								478,305 (84.38)		76,571 (73.72)		9673 (49.42)
		Drinker								88,570 (15.62)		27,303 (26.28)		9901 (50.58)
**Stress status^c^, n (%)**														
			Low to moderate							337,938 (59.61)		70,817 (68.18)		N/A
			High to severe							228,937 (40.39)		33,057 (31.82)		N/A
**Sadness and despair, n (%)**														
	Low to moderate									401,253 (70.78)		64,190 (61.8)		14,522 (74.19)
	High to severe									165,622 (29.22)		39,684 (38.2)		5052 (25.81)
**Exercise status, n (%)**														
							Not enough				496,475 (87.58)		87,510 (84.25)	N/A
							Enough				70,400 (12.42)		16,364 (15.75)	N/A
**Suicide thinking in the past year, n (%)**														
							No				482,613 (85.14)		80,561 (77.56)	17,652 (90.18)
							Yes				84,262 (14.86)		23,313 (22.44)	1922 (9.82)
**Screentime status, n (%)**														
				Low to moderate						165,382 (29.17)		27,569 (26.54)		994 (5.08)
				High to severe						401,493 (70.83)		76,305 (73.46)		18,580 (94.92)

^a^N/A: not applicable.

^b^BMI was divided into 4 groups according to the National Growth Charts: underweight (0-4th percentiles), normal (5th-84th percentiles), overweight (85th-94th percentiles), and obese (95th-100th percentiles).

^c^Stress was defined by receipt of mental health counseling owing to stress.

Both the initial training cohort and the external validation cohorts took into account socioeconomic backgrounds, such as household income and academic achievement, as well as risk behaviors such as alcohol consumption, smoking, and screen time. Additionally, factors that could potentially influence mental health, such as feelings of sadness and despair, were also considered. Inconsistencies or missing values in validation sets were addressed by implementing median imputation from the primary training data. Such thorough demographic incorporation bolsters our model performance, offering a nuanced understanding of suicidal thinking in adolescents.

### ML Model Results

**Figure 3:**
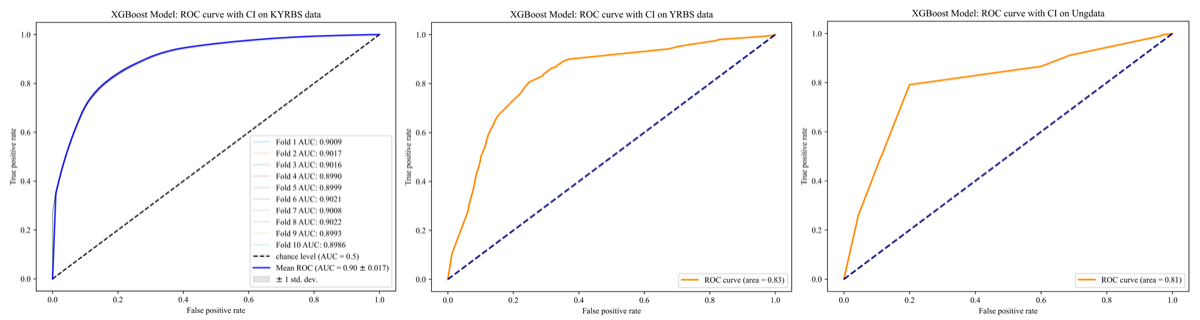
Receiver operating characteristic (ROC) curves with CIs for the Korea Youth Risk Behavior Web-based Survey (KYRBS), Youth Risk Behavior Survey (YRBS), and University National General Survey (Ungdata) of the XGBoost model.

Table S1 in Multimedia Appendix 1 and Table 2 present the process of hyperparameter tuning the XGBoost model and the evaluation of our models conducted on data sets from 3 distinct countries using 5 performance metrics. Notably, XGBoost emerged as the frontrunner among the 4 tested models by getting hyperparameters of booster: gbtree, eval_metric: logloss, learning_rate: 0.08, max_depth: 5, n_estimators: 350, scale_pos_weight: 2, subsample: 0.09 (Table S1 in Multimedia Appendix 1). When put to the training on the KYRBS data set from South Korea, with a 95% CI, the XGBoost model reported an AUROC of 90.06 (95% CI 89.97-90.16), sensitivity was 82.11 (95% CI 81.67-82.55), specificity was 82.16 (95% CI 81.68-82.63), accuracy was 82.13 (95% CI 82.01-82.26), and balanced accuracy was 82.13 (95% CI 82.01-82.26), consistently displaying superior results compared to the other 3 models. During the external validation assessment, the model evaluation was conducted without considering the 95% CI. For the external model validation, using the YRBS from the United States, the XGBoost model achieved an AUROC of 83.09%, sensitivity of 80.26%, specificity of 75.52%, accuracy of 76.58%, and balanced accuracy of 77.89%. For the external validation using the Ungdata from Norway, the XGBoost model achieved an AUROC of 81.27%, sensitivity of 79.19%, specificity of 80%, accuracy of 79.92%, and balanced accuracy of 79.60%. Across all data sets, XGBoost consistently outperformed all other models with the highest AUROC score, which was selected as the most optimal model (Figure 3).

**Table 2:** Various performance metrics for 4 different machine learning algorithms in the Korea Youth Risk Behavior Web-based Survey (KYRBS) data set for validation, the Youth Risk Behavior Survey (YRBS) data set for extra validation, and another external validation at the Norwegian nationwide University National General Survey (Ungdata) surveys data set.

Data set and model		AUROC^a^	Sensitivity	Specificity	Accuracy	Balanced accuracy
**South Korea, % (95% CI)**						
	XGBoost	90.06 (89.97-90.16)	82.11 (81.67-82.55)	82.16 (81.68-82.63)	82.13 (82.01-82.26)	82.13 (82.01-82.26)
	AdaBoost^b^	85.26 (85.14-85.38)	78.08 (77.94-78.21)	78.09 (77.96-78.22)	78.08 (77.95-78.21)	78.08 (77.95-78.21)
	LightGBM^c^	84.64 (84.53-84.75)	78.96 (78.88-79.04)	78.96 (78.88-79.04)	78.96 (78.88-79.04)	78.96 (78.88-79.04)
	Random forest	84.10 (83.99-84.21)	76.91 (76.73-77.10)	77.06 (76.98-77.15)	76.99 (76.86-77.11)	76.99 (76.86-77.11)
**United States, %**						
	XGBoost	83.09	80.26	75.52	76.58	77.89
	AdaBoost	82.13	76.75	77.06	76.99	76.91
	LightGBM	83.93	79.85	75.87	76.76	77.86
	Random forest	83.20	78.28	77.43	77.62	77.86
**Norway, %**						
	XGBoost	81.27	79.19	80.00	79.92	79.60
	AdaBoost	79.14	79.19	80.00	79.92	79.60
	LightGBM	84.14	77.98	81.95	81.87	79.97
	Random forest	79.23	79.50	79.50	79.55	79.97

^a^AUROC: area under the receiver operating characteristic.

^b^AdaBoost: adaptive boosting.

^c^LightGBM: light gradient-boosting machine.

### Feature Importance

Table 3 shows the feature importance derived from the XGBoost model, illustrating the relative contributions of each feature to predicting suicidal thinking. Notably, feelings of sadness and despair emerge as the most dominant predictor, accounting for 57.4% of the influence, followed by stress status at 19.8%. Subsequent factors include age (5.7%), household income (4%), academic achievement (3.4%), sex (2.1%), and others contributing less than 2% each.

**Table 3:** Feature importance of XGBoost.

Feature	Importance, %
Sadness and despair	57.4
Stress status	19.8
Age	5.7
Household income	4
Academic achievement	3.4
Sex	2.1
Smoking status	1.7
BMI	1.6
Alcohol consumption	1.3
Exercise status	1.3
Screentime status	1
Region	0.8

### SHAP Value

We addressed a deeper visual interpretation of the SHAP values within our ML model (Figure S2 in Multimedia Appendix 1) [29]. Figure S3 in Multimedia Appendix 1 provides a waterfall plot, distinctively showcasing the cumulative contribution of each feature to a single prediction. We interpreted individual predictions by starting from the initial estimate and sequentially incorporating the influence of each feature to reach the final prediction. E[f(x)] refers to the average predicted output of the model across the entire data set, providing insights into the model’s overall prediction tendency. The starting point of the illustration, denoted as E[f(x)]=0.83, represents the model’s average prediction for the given data set. Among the variables, sadness and despair stood out, boosting the prediction by 1.17 and ranking as the most influential factor. Conversely, stress status and sex reduced the prediction by 0.86 and 0.22, respectively. This visualization offers a clear insight into the profound influence each feature wields in predicting adolescent suicidal thinking. Our ML model notably underscores a substantial reliance on the sadness, despair, and stress status features (Figure S3 in Multimedia Appendix 1).

### Code Availability

Based on the results of the ML model, we established a web-based app for policy implementation or health system management to support in their decision-making process for cases involving suicidal thinking prediction use in adolescents [30]. An example of a web interface and the results are shown in Figure S4 in Multimedia Appendix 1. Custom code for the website is available on the web [31].

## Discussion

### Key Findings

Our research represents a pioneering machine-learning initiative for predicting suicidal thinking among adolescents. We sourced distinct data sets from South Korea (KYRBS), the United States (YRBS), and Norway (Ungdata). This provided comprehensive analysis related to socioeconomic indicators and key mental health influencers such as alcohol consumption, smoking status, and feelings of sadness and despair. Importantly, our findings highlight XGBoost as the optimal predictive model, achieving an AUROC of 88.6% with the KYRBS data set. External validation with the data from the United States and Norway yielded AUROCs of 82.9% and 83.6%, respectively. The most significant predictor of suicidal thinking was sadness and despair, with a feature influence of 61%, followed by stress status at 19.6%. Using SHAP values, we further emphasized the pivotal roles of sadness, despair, and stress in predicting suicidal thinking in adolescents. To enhance the practical application of our research, we have developed a web-based platform to visualize the prediction model, accompanied by a mobile interface, enhancing its accessibility and user experience. This dual-platform system provides a more methodical and analytical approach for the public to comprehend and manage potential suicidal concerns.

### Plausible Mechanism

The close relationship between feelings of sadness and despair and suicidal thinking in adolescents can be understood from various perspectives, encompassing biological and environmental factors [32]. During adolescence, the brain undergoes significant development, especially in the prefrontal cortex, which controls impulses and emotions [33]. Persistent sadness can interfere with adolescent brain development, resulting in a perpetual state of negative emotions. This increases their risk of suicidal thinking due to feelings of despair and impulsive actions [34].

The influence of external portrayals, be it from peers or the media, cannot be underestimated. When adolescents confront additional adversities, such as bullying, social isolation, or academic failures, these inherent stressors are amplified. Adolescents exposed to narratives associating despair with suicidal behaviors might inadvertently absorb these sentiments. This phenomenon, known as “suicide contagion,” postulates that exposure to others’ suicidal actions can reshape an individual’s perspective [35]. The confluence of these environmental stressors and limited emotional regulation capacities heightens their vulnerability, potentially leading them to view suicide as a viable solution to their emotional turmoil [36].

Furthermore, due to their developmental stage, many adolescents have not yet acquired the necessary emotional coping strategies [37]. When faced with intense stressors without these tools, some may come to view suicide as their only way to escape from increasingly desperate circumstances.

This emotional vulnerability is further compounded by physiological changes. The stress response, regulated by the hypothalamic-pituitary-adrenal axis, is intensified during adolescence [38]. Heightened sensitivity of the hypothalamic-pituitary-adrenal axis results in increased cortisol production in response to stress [39]. Chronic exposure to these elevated cortisol levels not only exacerbates feelings of sadness and despair but also directly contributes to an increased vulnerability to suicidal thoughts [40]. Understanding these relationships makes it evident that both the emotional responses induced by stress and the physiological effects of stress significantly influence the increased propensity for suicidal thinking during this critical phase of life.

### Strengths and Limitations

The limitations of this study should be stated. One primary concern pertains to the use of self-reported data, which exposes our results to potential biases, such as recall and social desirability. While this approach offers insights directly from the participants, such susceptibilities might skew the data and ultimately affect the model’s performance. It is also worth noting that the foundational training data were sourced predominantly from adolescents in South Korea [41,42]. This could amplify specific cultural or racial attributes distinctive to Korean adolescents. Equally important to note is that establishing a direct cause-and-effect relationship between significant risk factors and adolescent suicidal thinking remains elusive. This study, while expansive, does not determine if suicidal thinking is a cause or an effect of other risk factors. Further research is needed to unravel these complex interconnections. The potential for overfitting is another critical limitation to consider. Our comprehensive model, regardless of its use of 10-fold cross-validation, might inadvertently capture anomalies rather than genuine patterns [43]. Furthermore, our method for managing missing data, especially through median imputation, poses the risk of introducing unintended biases, which could impact the model’s performance [44]. Lastly, while predicting suicide attempts rather than suicide ideation may be crucial in suicide-related research, the low prevalence of suicide attempts presents limitations in constructing ML models: thus, we have developed predictive models for suicide ideation. With our predictive model, policy researchers, physicians, and community neighbors can develop individualized prevention strategies for these adolescents.

Despite these limitations, the strengths of this study are manifold. Notably, the SHAP value analysis highlights the importance of diverse demographic and behavioral indicators in understanding suicidal thinking among adolescents. This study provides nuanced insight into each feature’s unique influence and the model’s decision-making process [29]. The robustness of the model is also noteworthy, as evidenced by its uniform effectiveness across data sets spanning South Korea, the United States, and Norway. Such wide-reaching efficacy suggests the adaptability of the model to different cultural and demographic landscapes. Another strength lies in the real-world applicability of our research. The development of our advanced web-based platform and mobile interface marks a notable advancement in practical application. By offering a user-friendly interface tailored for both desktop and mobile users, we enhance accessibility and promote greater self-awareness about suicidal risk. This can encourage individuals to seek timely professional assistance or supportive resources, serving as a preventive measure against severe mental health crises [45].

### Clinical and Policy Implications

In light of the findings, several crucial policy implications emerge. Foremost, the significant role of sadness and despair as predictors underscores the necessity to prioritize mental health support for adolescents [46]. This prominence not only necessitates immediate interventions but also stresses the vital role of education and awareness initiatives, targeting both risk behaviors and associated mental health ramifications. Early identification of suicidal thinking is paramount [47]. Recognizing these indications enables health care professionals to initiate early intervention strategies. This preemptive approach should include tailored counseling, support group engagements, or intensive therapeutic interventions. This can prevent the progression toward actual suicide attempts, which might be driven by mixed emotions or even an intent just to signal distress [48]. Moreover, there is a pressing need to bolster educational and awareness campaigns concerning suicide. Such campaigns serve to equip adolescents with the tools and knowledge necessary, encouraging them to navigate challenges related to risky behaviors and maintain positive mental health perspectives [49].

### Conclusion

In addressing the pressing global concern of adolescent suicide, this study used ML to offer novel insights into preemptive detection. By integrating diverse data sets across 3 nations, the study highlighted the superiority of the XGBoost model in predicting suicidal thinking, achieving remarkable AUROCs of 90.06% (95% CI 89.97-90.16; KYRBS from South Korea; discovery), 83.09% (YRBS from the United States; extra validation), and 81.27% (Ungdata from Norway; extra validation). Our findings emphasize the significant role of emotional health indicators in predicting suicidal thinking among adolescents. Specifically, sadness and despair proved to be the most influential predictors, followed by stress status and age. Through our robust, cross-culturally validated model and its accessibility through web-based platforms, we underscore the potential for timely interventions and offer a promising blueprint for future mental health strategies and preventive measures for at-risk adolescents.

## Appendix

Multimedia Appendix 1 Study population; SHAP value of XGBoost model; SHAP waterfall plot; deployed web-based application to provide suicidal thinking prediction among adolescents; process of hyperparameter tuning for the XGBoost model on KYRBS data set; and transparent reporting of a multivariable prediction model for individual.

## Abbreviations

AdaBoost: adaptive boosting
AUROC: area under the receiver operating characteristic
KYRBS: Korea Youth Risk Behavior Web-based Survey
LightGBM: light gradient-boosting machine
ML: machine learning
SHAP: Shapley additive explanations
TRIPOD: Transparent Reporting of a Multivariable Prediction Model for Individual Prognosis or Diagnosis
Ungdata: University National General Survey
YRBS: Youth Risk Behavior Survey

## Data Availability

The data sets generated during and/or analyzed during this study are available from the corresponding author upon reasonable request. The study protocol and statistical code are available from DKY. The data set can be accessed from the Korean Disease Control and Prevention Agency, US Centers for Disease Control and Prevention, and Norwegian Social Research Institute (NOVA) through a data use agreement.
