# Supplementary material for: Machine Learning–Based Prediction of Suicidal Thinking in Adolescents by Derivation and Validation in 3 Independent Worldwide Cohorts: Algorithm Development and Validation Study
Source: J Med Internet Res. 2024 May 17;26:e55913. doi: 10.2196/55913 (PMC11143390; doi:10.2196/55913)
Supplement: Multimedia Appendix 1 [file jmir_v26i1e55913_app1.docx]

**Figure S1**. Study population. KYRBS: Korea Youth Risk Behavior Web-based Survey; Ungdata: Norwegian nationwide Ungdata surveys; YRBS: Youth Risk Behavior Survey of United States adolescent.


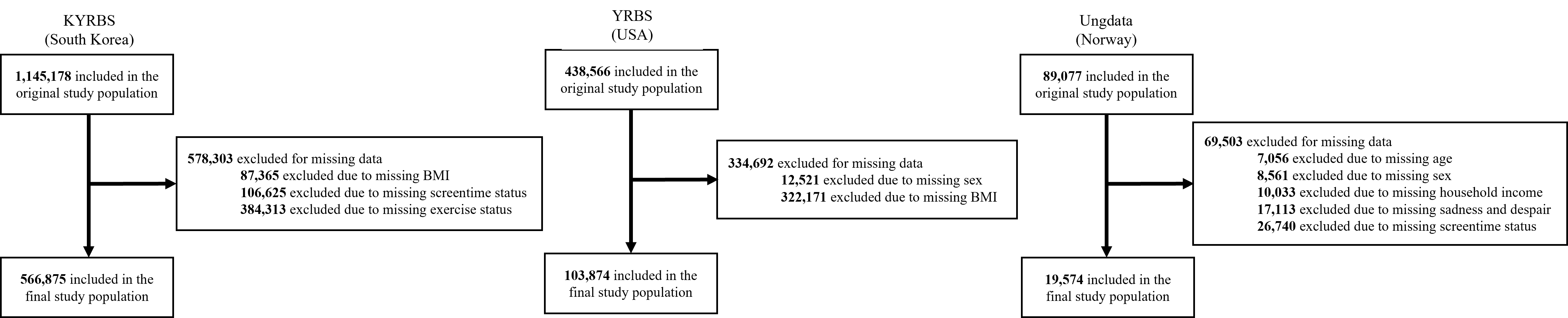


**Figure S2**. Shapley Additive Explanations (SHAP) value of Extreme Gradient Boosting (XGBoost) model.


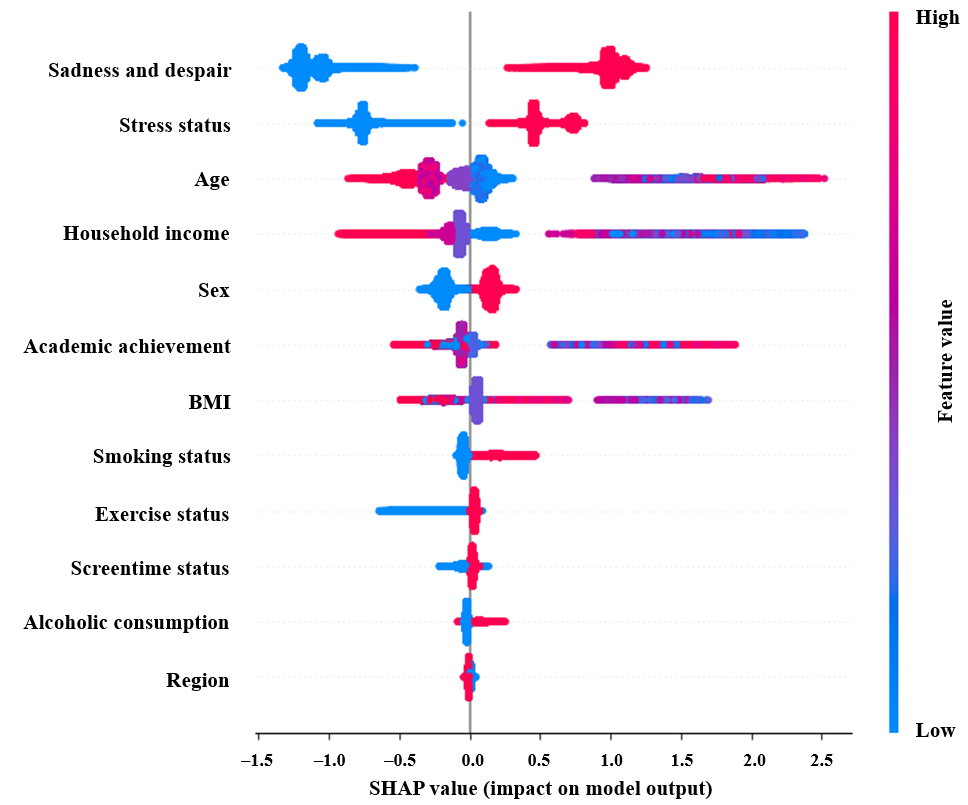


**Figure S3**. Shapley Additive Explanations (SHAP) waterfall plot illustrating the contribution of each feature to the prediction. f(x) represents the model’s output prediction for a specific data point. Meanwhile, E[f(x)] denotes the average predicted output of model across the entire dataset, providing insights into the model’s overall prediction tendency.


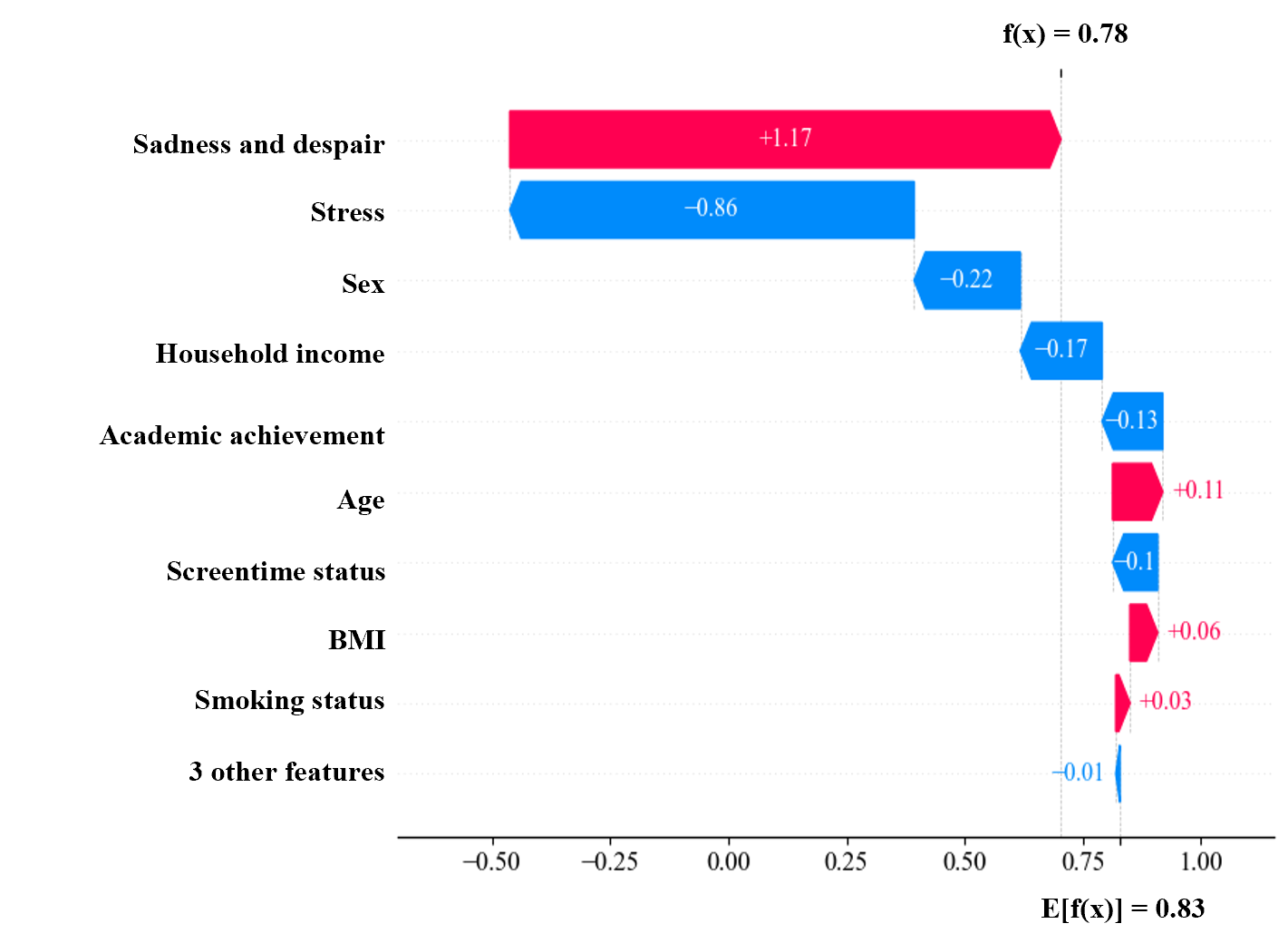


**Figure S4**. Deployed web-based application to provide suicidal thinking prediction among adolescents: a web interface of user to enter information and the prediction results with the probability of suicidal thinking among adolescents.

**
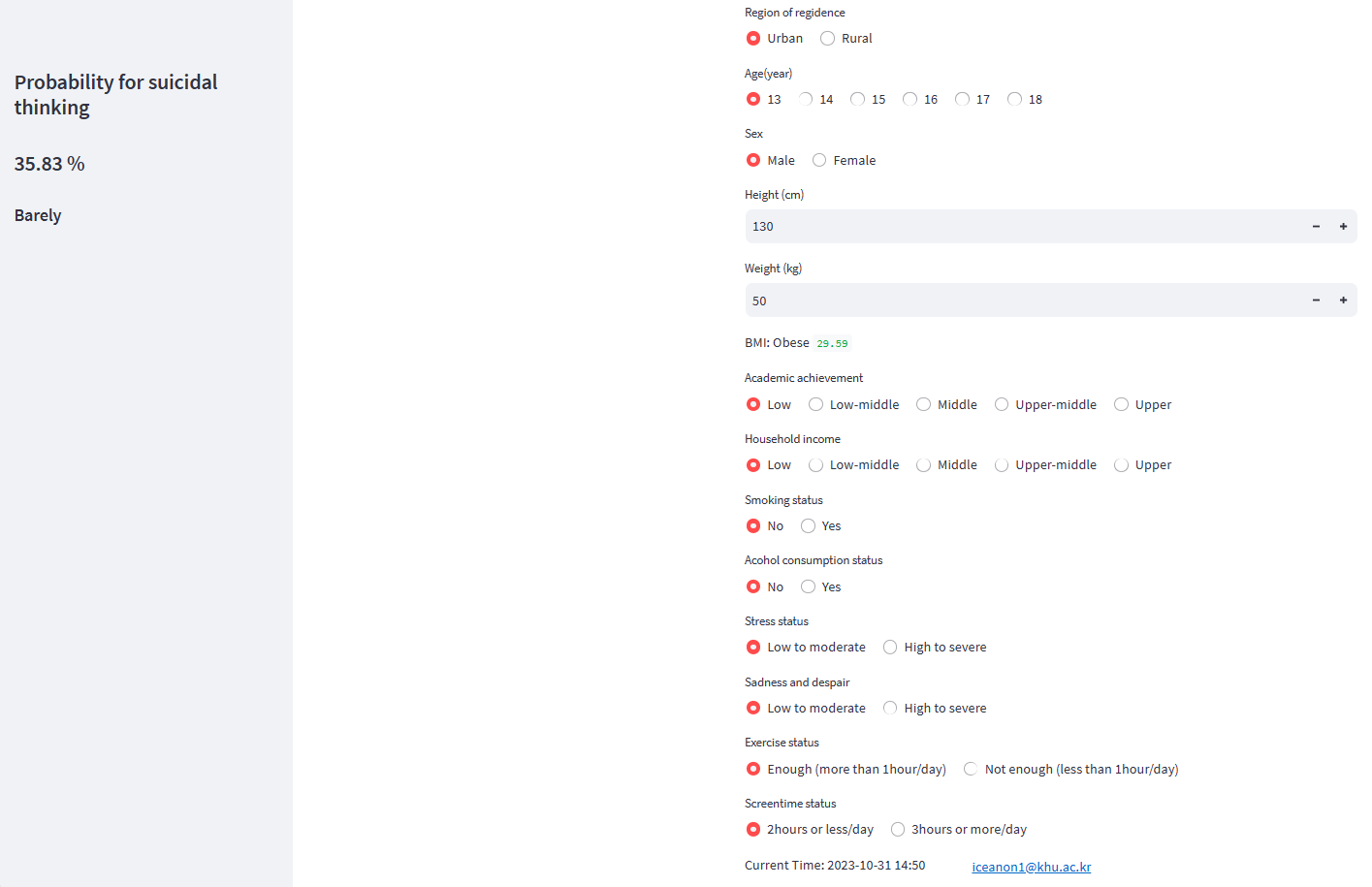
**

**Table S1**. Process of hyperparameter tuning for the Extreme Gradient Boosting (XGBoost) model on Korea Youth Risk Behavior Web-based Survey (KYRBS) data set. AUROC: area under the receiver operating characteristic curve.

| Hyperparameter | AUROC | Sensitivity | Specificity | Accuracy | Balanced accuracy |
| --- | --- | --- | --- | --- | --- |
| booster: Gbtree  eval_metric: logloss  learning_rate: 0.08  max_depth: 4  n_estimators: 115  scale_pos_weight: 6  subsample: 0.09 | 87.79 (87.67, 87.90) | 80.78 (80.34, 81.22) | 79.74 (79.19, 80.30) | 80.26 (80.16, 80.37) | 80.26 (80.16, 80.37) |
| booster: gbtree,  eval_metric: logloss  learning_rate: 0.08  max_depth: 5  n_estimators: 150  scale_pos_weight: 2  subsample: 0.09 | 89.40 (89.30, 89.50) | 82.16 (81.74, 82.58) | 81.36 (80.89, 81.82) | 81.76 (81.65, 81.87) | 81.76 (81.65, 81.87) |
| booster: gbtree  eval_metric: logloss  learning_rate: 0.3  max_depth: 8  n_estimators: 250  scale_pos_weight: 2  subsample: 0.09 | 89.96 (89.86, 90.06) | 82.71 (82.13, 83.30) | 81.56 (80.92, 82.20) | 82.14 (82.03, 82.25) | 82.14 (82.03, 82.25) |
| booster: gbtree  eval_metric: logloss  learning_rate: 0.08  max_depth: 5  n_estimators: 300  scale_pos_weight: 2,  subsample: 0.09 | 90.02 (89.93, 90.12) | 82.08 (81.52, 82.64) | 82.15 (81.58, 82.72) | 82.12 (81.99, 82.24) | 82.12 (81.99, 82.24) |
| **booster: gbtree**  **eval_metric: logloss**  **learning_rate: 0.08**  **max_depth: 5**  **n_estimators: 350**  **scale_pos_weight: 2**  **subsample: 0.09** | **90.06 (89.97, 90.16)** | **82.11 (81.67, 82.55)** | **82.16 (81.68, 82.63)** | **82.13 (82.01, 82.26)** | **82.13 (82.01, 82.26)** |

**Table S2**. Transparent reporting of a multivariable prediction model for individual.

| **Section/Topic** | **Item** |  | **Checklist Item** | **Page** |
| --- | --- | --- | --- | --- |
| **Title and abstract** | | | | |
| Title | 1 | D;V | Identify the study as developing and/or validating a multivariable prediction model, the target population, and the outcome to be predicted. | 1 |
| Abstract | 2 | D;V | Provide a summary of objectives, study design, setting, participants, sample size, predictors, outcome, statistical analysis, results, and conclusions. | 3-4 |
| **Introduction** | | | | |
| Background and objectives | 3a | D;V | Explain the medical context (including whether diagnostic or prognostic) and rationale for developing or validating the multivariable prediction model, including references to existing models. | 5 |
|  | 3b | D;V | Specify the objectives, including whether the study describes the development or validation of the model or both. | 5 |
| **Methods** | | | | |
| Source of data | 4a | D;V | Describe the study design or source of data (e.g., randomized trial, cohort, or registry data), separately for the development and validation data sets, if applicable. | 6,  Figure 1 |
|  | 4b | D;V | Specify the key study dates, including start of accrual; end of accrual; and, if applicable, end of follow-up. | NA |
| Participants | 5a | D;V | Specify key elements of the study setting (e.g., primary care, secondary care, general population) including number and location of centres. | 6 |
|  | 5b | D;V | Describe eligibility criteria for participants. | 6 |
|  | 5c | D;V | Give details of treatments received, if relevant. | NA |
| Outcome | 6a | D;V | Clearly define the outcome that is predicted by the prediction model, including how and when assessed. | 6-8 |
|  | 6b | D;V | Report any actions to blind assessment of the outcome to be predicted. | NA |
| Predictors | 7a | D;V | Clearly define all predictors used in developing or validating the multivariable prediction model, including how and when they were measured. | 6-8 |
|  | 7b | D;V | Report any actions to blind assessment of predictors for the outcome and other predictors. | NA |
| Sample size | 8 | D;V | Explain how the study size was arrived at. | 6-8 |
| Missing data | 9 | D;V | Describe how missing data were handled (e.g., complete-case analysis, single imputation, multiple imputation) with details of any imputation method. | 6-8,  Figure 1 |
| Statistical analysis methods | 10a | D | Describe how predictors were handled in the analyses. | 6-8 |
|  | 10b | D | Specify type of model, all model-building procedures (including any predictor selection), and method for internal validation. | 6-8 |
|  | 10c | V | For validation, describe how the predictions were calculated. | 6-8 |
|  | 10d | D;V | Specify all measures used to assess model performance and, if relevant, to compare multiple models. | 6-8 |
|  | 10e | V | Describe any model updating (e.g., recalibration) arising from the validation, if done. | 6-8 |
| Risk groups | 11 | D;V | Provide details on how risk groups were created, if done. | NA |
| Development vs. validation | 12 | V | For validation, identify any differences from the development data in setting, eligibility criteria, outcome, and predictors. | 6-8 |
| **Results** | | | | |
| Participants | 13a | D;V | Describe the flow of participants through the study, including the number of participants with and without the outcome and, if applicable, a summary of the follow-up time. A diagram may be helpful. | 9,  Figure 1 |
|  | 13b | D;V | Describe the characteristics of the participants (basic demographics, clinical features, available predictors), including the number of participants with missing data for predictors and outcome. | 9,  Figure 1,  Table 1 |
|  | 13c | V | For validation, show a comparison with the development data of the distribution of important variables (demographics, predictors and outcome). | 9,  Table 1 |
| Model development | 14a | D | Specify the number of participants and outcome events in each analysis. | 9,  Table 1 |
|  | 14b | D | If done, report the unadjusted association between each candidate predictor and outcome. | NA |
| Model specification | 15a | D | Present the full prediction model to allow predictions for individuals (i.e., all regression coefficients, and model intercept or baseline survival at a given time point). | 9-11 |
|  | 15b | D | Explain how to use the prediction model. | 9-11,  Figure 2 |
| Model performance | 16 | D;V | Report performance measures (with CIs) for the prediction model. | 9-11,  Figure 3 |
| Model-updating | 17 | V | If done, report the results from any model updating (i.e., model specification, model performance). | 10-11,  Figure 3, S1, S2 |
| **Discussion** | | | | |
| Limitations | 18 | D;V | Discuss any limitations of the study (such as nonrepresentative sample, few events per predictor, missing data). | 10-11 |
| Interpretation | 19a | V | For validation, discuss the results with reference to performance in the development data, and any other validation data. | 9-12 |
|  | 19b | D;V | Give an overall interpretation of the results, considering objectives, limitations, results from similar studies, and other relevant evidence. | 9-12 |
| Implications | 20 | D;V | Discuss the potential clinical use of the model and implications for future research. | 11-12 |
| **Other information** | | | | |
| Supplementary information | 21 | D;V | Provide information about the availability of supplementary resources, such as study protocol, Web calculator, and data sets. | 16,19 |
| Funding | 22 | D;V | Give the source of funding and the role of the funders for the present study. | 18 |

prognosis or diagnosis (TRIPOD) statement

*Items relevant only to the development of a prediction model are denoted by D, items relating solely to a validation of a prediction model are denoted by V, and items relating to both are denoted D;V. We recommend using the TRIPOD Checklist in conjunction with the TRIPOD Explanation and Elaboration.
